# The Relationship Between Gastrointestinal Comorbidities, Clinical Presentation and Surgical Outcome in Patients with DCM: Analysis of a Global Cohort

**DOI:** 10.3390/jcm9030624

**Published:** 2020-02-26

**Authors:** Aria Nouri, Jetan H. Badhiwala, So Kato, Hamed Reihani-Kermani, Kishan Patel, Jefferson R. Wilson, Insa Janssen, Joseph S. Cheng, Karl Schaller, Enrico Tessitore, Michael G. Fehlings

**Affiliations:** 1 Department of Neurosurgery, University of Geneva, 1205 Geneva, Switzerland; arianouri9@gmail.com (A.N.); InsaKatrin.Janssen@hcuge.ch (I.J.); Karl.Schaller@hcuge.ch (K.S.); enrico.tessitore@hcuge.ch (E.T.); 2 Department of Neurosurgery, University of Cincinnati College of Medicine, Cincinnati, OH 45267-0515, USA; chengj6@ucmail.uc.edu; 3 Division of Neurosurgery, University of Toronto, Toronto, ON M5S 1A1, Canada; jetan.badhiwala@gmail.com (J.-H.B.); jeffersonwilson7@gmail.com (J.R.W.); 4 Department of Orthopaedic Surgery, University of Tokyo, Tokyo 100068, Japan; sokato34@gmail.com; 5 Neuroscience Research Center, Kerman University of Medical Sciences, Kerman, Iran; h_reihani@hotmail.com; 6 Department of Neurosurgery, Yale University, New Haven, CT 06520-8082, USA; kishan.patel@yale.edu

**Keywords:** cervical spondylotic myelopathy (CSM), prospective, multicenter, anterior, posterior

## Abstract

Degenerative cervical myelopathy (DCM) is the most common cause of spinal cord impairment in adults, presenting most frequently in patients 50 years or older. Gastrointestinal comorbidities (GICs) commonly occur in this group; however, their relationship with DCM has not been thoroughly investigated. It is the objective of the present study to investigate the difference between patients with or without GICs who are surgically treated for DCM. A cohort of 757 patients with clinical data and 458 with magnetic resonance imaging (MRI) data from the AOSpine North America and AOSpine International studies on DCM was evaluated. GICs were obtained at presentation and included gastric, intestinal, hepatic, and pancreatic conditions. Patients were dichotomized into 2 groups: those with GICs and those without GICs. Both clinical and MRI presentation, as well as baseline neurological and functional status, were compared. Neurological and functional outcomes at 2-year follow-up were also compared. GICs were present in 121 patients (16%). These patients were less commonly male (48.76% vs. 65.4%, *p* = 0.001) and were slightly less neurologically impaired based on the Nurick grade (3.05 ± 1.10 vs. 3.28 ± 1.16, *p* = 0.044) but not based on mJOA (12.74 ± 2.62 vs. 12.48 ± 2.76, *p* = 0.33). They also had a worse physical health score (32.80 ± 8.79 vs. 34.65 ± 9.38 *p* = 0.049), worse neck disability (46.31 ± 20.04 vs. 38.23 ± 20.44, *p* < 0.001), a lower prevalence of upper motor neuron signs (hyperreflexia, 70.2% vs. 78.9%, *p* = 0.037; Babinski’s sign 24.8% vs. 37.3%, *p* = 0.008), and a higher rate of psychiatric comorbidities (31.4% vs. 10.4%, *p* < 0.0001). On MRI, GIC patients less commonly exhibited signal intensity changes (T2 hyperintensity, 49.2% vs. 75.6%, *p* < 0.001; T1 hypointensity, 9.7% vs. 21.1%, *p* = 0.036), and had a lower number of T2 hyperintensity levels (0.82 ± 0.98 vs. 1.3 ± 1.11, *p* = 0.001). There was no difference in surgical outcome between the groups. DCM patients with GICs are more likely to be female and have significantly more general health impairment and neck disability. However, these patients have less clinical and MRI features typical of more severe neurological impairment. This constellation of symptoms is considerably different than those typically observed in DCM, and it is therefore plausible that nutritional factors may contribute to this unique observation.

## 1. Introduction

Degenerative cervical myelopathy is the most common cause of spinal cord impairment in industrialized countries and can lead to significant neurological and functional dysfunction, as well as reduced quality of life [1]. The underlying pathology is heterogeneous and can include intervertebral disc disease, arthritic changes, hypertrophy and/or ossification of the spinal canal ligaments, and spondylolisthesis, ultimately leading to spinal cord injury through static and dynamic injury mechanisms [1]. Depending on the number of cervical levels involved, the degree of cord compression, and the natural history, patients present with a wide-ranging spectrum of clinical manifestations [2,3]. Symptoms include hyperreflexia, weakness, numbness, and loss of proprioception/balance, and clinical signs, such as Hoffmann’s sign, Babinski reflex, Lhermitte’s phenomenon, ankle clonus, inverted brachioradialis reflex, and Romberg’s sign, which may be elicited on clinical examination [2,4,5]. Neurophysiological examination may indicate changes in motor and sensory evoked potentials; MRI signal intensity changes on T2 and T1 may highlight injury to the spinal cord. These various clinical factors and examinations have been used to assess degree of neurological impairment and surgical outcome. However, relatively little research has been undertaken to assess how comorbidities, such as gastrointestinal disease, impact baseline neurological status, and recovery potential in patients undergoing surgical treatment. 

Gastrointestinal comorbidities (GICs) have the potential to influence the presentation and recovery of patients with myelopathy in a number of ways. For example, GICs can result in malnutrition (such as hypocupremia), anemia through blood loss, and vitamin B12 (B12) deficiency—all of which may impact spinal cord function or surgical recovery [6,7]. With regard to B12 deficiency, it has been recently suggested that vitamin B12 (B12) deficiency may be a common and under-recognized comorbidity in patients with DCM [8], and is also a differential diagnosis [9,10]. It has also been shown that anemia is related to higher surgical morbidity, worse neurological status at baseline and neurological outcomes, higher rates of medical complications, and raises the risk of complications by increasing the probability that a patient will require an allogeneic RBC infusion [11,12,13]. Other studies have shown that malnutrition increases 90-day major medical complications, 1-year mortality, and is a predictor of increased infection and wound dehiscence rates after lumbar spine surgery [14].

Given that identification of sequalae of GICs may have an important impact on the clinical management of DCM patients, it is the objective of the present paper to evaluate the influence of GICs on baseline neurological function and surgical outcomes for treatment of DCM. 

## 2. Methods

### 2.1. Study Data

The combined AOSpine study cohort comprises 757 patients (AOSpine North American study, *n* = 278; AOSpine International Study, *n* = 479) [15,16]. The North American study was conducted between 2005 and 2007 and included 12 North American sites (11 USA, 1 Canada); the International study was conducted between 2007 and 2011 and included 16 global sites comprising 4 regions (North America, Latin America, Asia, and Europe). The primary study objective was to assess the safety and efficacy of surgical treatment for DCM and was previously reported [15,16]. Adult patients (≥18 years of age) were included if they had clinical signs and symptoms of myelopathy that were confirmed via imaging. Patients were excluded if they had an active infection, neoplastic disease, rheumatoid arthritis, ankylosing spondylitis, previous surgery, or concomitant signs of lumbar stenosis. Patient clinical data, general health (SF-36) [17], Neck Disability Index (NDI) [18], and neurological function (modified Japanese Association score [mJOA] [19] and Nurick grade [20] were assessed. The pain subscore of NDI, which ranges from 0 to 5, was assessed to specifically evaluate pain. GICs were recorded non-specifically as present or absent and included potential gastric, hepatic, pancreatic, and intestinal comorbidities. Research ethics board approval was given at each participating center, and external monitors were used to visit the sites. 

### 2.2. MRI Data

MRI (1.5T or 3T) acquisitions were performed according to local protocols (no standardized protocols were used), and typically included axial and sagittal T2-weighted and sagittal T1-weighted images. DICOM (Digital Imaging and Communications in Medicine) and conventional image formats (JPEG, TIFF) were reviewed. DICOMs were reviewed using Osirix (www.osirix-viewer.com; Pixmeo, Geneva, Switzerland). MRIs were available for 458 patients, and the prevalence and spectrum of DCM pathology were previously published [21]. MRIs were assessed for the presence and absence of specific pathologies (e.g., isolated disc pathology, spondylolisthesis), for the presence of T2 signal hyperintensity, and T1 signal hypointensity changes. Signal intensity changes on T2 and T1 were reviewed by 3 raters, and the relationship between these changes and clinical presentation, as well as surgical outcome, were previously reported [2]. Therein, inter-rater reliability for signal changes was reported as being in substantial agreement for T2 hyperintensity (Fleiss Kappa: 0.60), and in fair agreement for T1 hypointensity (Fleiss Kappa: 0.31). 

### 2.3. Statistical Analysis

Statistical analysis was performed with SPSS (version 25.0, IBM, Armonk, NY, USA). Patients with DCM were separated into groups comprising those with or without GICs. Continuous variables are presented as means and were compared using independent t-tests. Categorical variables are presented as proportions and were assessed using Chi square. A last observation carry-forward approach was used to impute missing data for follow-up at 2 years. Measures of neurological and functional impairment between patients with and without GICs were compared at baseline and 2-year follow-up (mean difference from baseline) using independent t-tests. The baseline pain subscore of NDI was compared using an independent t-test. As a sensitivity analysis, between-group comparisons of change in mJOA, Nurick grade, NDI, and SF-36 physical component summary (PCS) and mental component summery (MCS) from baseline were made with the use of mixed-effects models for repeated measures. Fixed effects for the presence of GICs (GICs vs. no GICs), time (1 year, 2 year), and time x GIC interaction were included. Comparisons of least-squares means between groups at each time point were performed using the appropriate contrasts within the mixed-effect models. 

## 3. Results

There were 121 patients (16%) with GICs and 636 patients (84%) without GICs (Table 1). GIC patients were less commonly male (48.76% vs. 65.4%, *p* = 0.001) and were on average 2 years older than patients without GICs (57.98 ± 10.21 vs. 56.04 ± 12.10, *p* = 0.065); however, this did not reach statistical significance. Neurologically, GIC patients were marginally less impaired than patients without GICs (Nurick grade, 3.05 ± 1.10 vs. 3.28 ± 1.16, *p* = 0.044; mJOA, 12.74 ± 2.62 vs. 12.48 ± 2.76, *p* = 0.33) but had a higher rate of psychiatric comorbidities (31.4% vs. 10.4%, *p* < 0.0001). Patients with GICs also had worse physical disability (SF-36 PCS, 32.80 ± 8.79 vs. 34.65 ± 9.38, *p* = 0.049) and worse neck disability (NDI, 46.31 ± 20.04 vs. 38.23 ± 20.44, *p* < 0.001), but a lower prevalence of upper motor signs (hyperreflexia, 70.2% vs. 78.9%, *p* = 0.037; Babinski’s sign 24.8% vs. 37.3%, *p* = 0.008). Duration of symptoms was similar for patients with and without GICs. The baseline NDI pain subscore was significantly worse in patients with GICs than those without (2.27 ± 1.32 vs. 1.75 ± 1.31, *p* < 0.001).

On MRI, patients with GICs less commonly exhibited signal intensity changes (T2 hyperintensity, 49.2% vs. 75.6%, *p* < 0.001; T1 hypointensity, 9.7% vs. 21.1%, *p* = 0.036) and had a lower number of T2 hyperintensity levels (0.82 ± 0.98 vs. 1.3 ± 1.11, *p* = 0.001) than patients without GICs. However, there were no differences in the number of compressed levels or the prevalence of combined anterior–posterior compression.

There were no differences in neurological or functional outcomes at 2-year follow-up between patients with or without GICs (Table 2 and Table 3). 

## 4. Discussion

Gastrointestinal conditions are common in the elderly, and therefore, it is not surprising that GICs were prevalent in 16% of patients with DCM. What is clear from this study is that patients with GICs represent a unique cohort that is quite different from the typical DCM patient: (1) they are more commonly female, despite the fact that prevalence of DCM among males is greater among reported studies (1), (2) almost a third of patients have psychiatric comorbidities, a much higher prevalence than otherwise expected, (3) patients had a large discrepancy between their general health measure score and NDI vs. neurological measures, showing significantly increased general health and neck disability but milder neurological impairment, and (4) GIC patients showed significantly lower MRI evidence of cord injury, despite having only subtle differences in neurological function. Given the large sample of patients with GICs (*n* = 121) in the cohort and the substantial deviance of clinical presentation in multiple dimensions (i.e., demographic, general health, neck pain, and objective findings of neurologic injury), it is clear that the presence of GICs is influential, but due to its broad categorization, it is challenging to account for specific factors.

Generally, GICs can result in a number of potential conditions, including anemia (due to blood loss), as well as malnutrition and vitamin deficiencies (due to GI resections or inflammatory conditions) that are essential to spinal cord function [6,7,22]. Anemia is usually easily identified through preoperative screening, and its preoperative presence should be managed prior to surgery to avoid complications. Indeed, it has been previously shown using the NSQIP database that preoperative anemia is an independent risk factor for complications, the need for perioperative blood transfusion, return to the operating room, and extended length of stay after cervical surgery [12,23]. From a different perspective, it has also been proposed through animal studies that spinal cord compression may result in irregular nervous stimulation of the stomach, a phenomenon termed neck-stomach syndrome [24]. However, this connection remains largely unexplored.

The potential role of nutritional or vitamin deficiencies in DCM has not been adequately investigated, and therefore, it is unclear how these patients would present. In general, it is known that lack of nutritional factors is contributory to the health of intervertebral discs, as the avascular nature of discs and reliance on diffusion renders them susceptible to injury due to undernutrition. In particular, nutritional levels must exceed a critical threshold for the cells to remain viable and active [25].

Two nutritional factors that may be specifically relevant to neurological function include B12 deficiency and copper deficiency (hypocupremia). Deficiency of either of these can result in both myelopathy and anemia [6,7]. Copper deficiency is rare, typically manifests due to high zinc intake, gastric resection, and malabsorption, and in a majority of cases treatment does not reverse myelopathic injury [6]. In contrast, B12 deficiency is much more common: It has been estimated that subclinical or clinical deficiency exists in up to 20% of elderly patients [26]. Further, clinical manifestation of B12 deficiency can mimic DCM and present with T2 cord hyperintensity. B12 deficiency has been reported to occur concomitantly with DCM [27,28,29], as well as in patients with suspected DCM—but underlying SACD—who experienced a resolution of symptoms after B12 administration [9,10,30]. B12 deficiency is most commonly due to pernicious anemia, bowel resection, inflammatory bowel disorders, liver disease, or gastric atrophy [31,32]. Unfortunately, without lab work to corroborate this, this remains speculative. However, B12 deficiency is also known to cause cognitive impairment and neuropsychiatric disease [33] and could be responsible for the high level of psychiatric comorbidities observed amongst patients with GICs in the present analysis. The relationship between psychiatric and gastrointestinal comorbidities, as well as other somatic symptoms such as back pain, has been previously reported [34,35]. For example, a recent study on irritable bowel syndrome concluded that psychiatric factors could contribute to predisposition, precipitation, and perpetuation of IBS symptoms [36]. Such findings suggest a potential explanation for the significantly different levels of neck disability between the two groups, as it is plausible that a higher rate of psychiatric comorbidities contributed to the higher rate of non-objectifiable symptoms. It also suggests that perhaps the high level of psychiatric symptoms is the reason for this different population clinical phenotype.

Overall, the findings suggest that patients with GICs were less commonly severely neurologically impaired. This is evidenced by the lower prevalence of objective upper motor signs (Babinski’s reflex, hyperreflexia) and MRI evidence typical of more severely impaired patients (T1 hypointensity). Despite these and a marginally lower Nurick grade, there was no statistically significant difference in surgical outcomes between patients with or without GICs.

### Limitations

A clear limitation to this study is the nonspecific nature of having classified patients into a single group of gastrointestinal comorbidities. It would have been preferable to know specific diagnoses; however, these data were not available. Furthermore, given that the main study was not focused on gastrointestinal disease, we may have not captured an accurate population prevalence. Due to this, caution needs to be taken in interpreting the results, as false positive relationships are possible. Further, we have hypothesized that the unique differences observed here are possibly due to nutritional deficiencies; however, further work is needed to corroborate this. Lastly, because MRI data were derived from multiple global sites, there was no standardized protocol used to obtain MRIs.

## 5. Conclusions

Patients with GICs represent a unique cohort that is different from typical DCM patients: (1) they are more commonly female, (2) almost a third of patients have psychiatric comorbidities, and (3) they have worse general health and NDI findings, but less severe neurological deficits and MRI evidence of neurological impairment. This constellation of symptoms is considerably different than those typically observed in DCM; it is therefore plausible that nutritional factors that frequently manifest in elderly patients may contribute to this unique observation.

## Figures and Tables

**Table 1:** Patient demographics and clinical and MRI presentation.

Clinical	Gastrointestinal Comorbidity	Non-Gastrointestinal Comorbidity	*p*-Value
Age	57.98 ± 10.21 (*n* = 121)	56.04 ± 12.10 (*n* = 636)	0.065
Sex (Male)	48.76% (*n* = 59/121)	65.4% (*n* = 220/636)	**0.001**
Duration of Symptoms	26.24 ± 38.22 (*n* = 121)	26.64 ± 39.14 (*n* = 636)	0.918
Psychiatric Comorbidities	31.4% (*n* = 38/121)	10.4% (*n* = 66/636)	**<0.0001**
mJOA	12.74 ± 2.62 (*n* = 120)	12.48 ± 2.76 (*n* = 623)	0.33
Nurick	3.05 ± 1.10 (*n* = 121)	3.28 ± 1.16 (*n* = 636)	**0.044**
NDI	46.31 ± 20.04 (*n* = 100)	38.23 ± 20.44 (*n* = 560)	**0.0003**
SF-36 PCS	32.80 ± 8.79 (*n* = 116)	34.65 ± 9.38 (*n* = 618)	**0.049**
SF-36 MCS	38.47 ± 14.01 (*n* = 116)	40.57 ± 13.43 (*n* = 618)	0.124
**Clinical Signs and Symptoms (*n* = 756)**			
Numb Hands	92.6% (*n* = 112/121)	88.2% (*n* = 560/635)	0.16
Clumsy Hands	71.9% (*n* = 87/121)	74.6% (*n* = 474/635)	0.53
Impairment of Gait	74.4% (*n* = 90/121)	75.4% (*n* = 479/635)	0.81
Bilateral arm paresthesia	61.2% (*n* = 74/121)	55.7% (*n* = 354/635)	0.27
Lhermitte’s Phenomena	29.8% (*n* = 36/121)	26.0% (*n* = 165/635)	0.39
Weakness	83.5% (*n* = 101/121)	82.2% (*n* = 522/635)	0.74
Corticospinal motor deficits	62.0% (*n* = 75/121)	62.5% (*n* = 397/635)	0.91
Atrophy of hand intrinsic muscles	35.5% (*n* = 43/121)	35.9% (*n* = 228/635)	0.94
Hyperreflexia	70.2% (*n* = 85/121)	78.9% (*n* = 501/635)	**0.037**
Hoffmann’s Sign	58.7% (*n* = 71/121)	62.7% (*n* = 398/635)	0.41
Babinski’s sign	24.8% (*n* = 30/121)	37.3% (*n* = 237/635)	**0.008**
Lower limb spasticity	40.5% (*n* = 49/121)	47.9% (*n* = 304/635)	0.14
Broad-based, unstable gait	55.4% (*n* = 67/121)	59.1% (*n* = 375/635)	0.45
**MRI**			
Anterior–Posterior Compression	53.7% (*n* = 36/67)	59.8% (*n* = 234/391)	0.35
Number of Levels Compressed	2.91 ± 1.25 (*n* = 67)	3.16 ± 1.24 (*n* = 391)	0.13
T2 Hyperintensity (*n* = 446)	49.2% (*n* = 32/65)	75.6% (*n* = 288/381)	**<0.0001**
T1 Hypointensity (*n* = 422)	9.7% (*n* = 6/62)	21.1% (*n* = 76/360)	**0.036**
Levels of T2 Hyperintensity	0.82 ± 0.98 (*n* = 65)	1.3±1.11 (*n* = 381)	**0.001**

NDI, Neck Disability Index; MRI, magnetic resonance imaging; mJOA, modified Japanese Orthopaedic Association scale; PCS, physical component summary; MCS, mental component summary.

**Table 2:** Surgical outcome at 2-years follow-up.

Outcome at 2-years (Mean Difference)	Gastrointestinal Comorbidity	Non-Gastrointestinal Comorbidity	*p*-Value
mJOA	2.96 ± 2.72 (*n* = 112)	2.66 ± 2.93 (*n* = 575)	0.30
Nurick	−1.55 ± 1.39 (*n* = 113)	−1.39 ± 1.47 (*n* = 586)	0.30
NDI	−10.84 ± 20.78 (*n* = 93)	−12.03 ± 19.04 (*n* = 512)	0.59
SF-36 PCS	5.95 ± 10.84 (*n* = 108)	5.64 ± 10.42 (*n* = 568)	0.78
SF-36 MCS	4.61 ± 15.66 (*n* = 108)	5.69 ± 13.74 (*n* = 568)	0.50

**Table 3:** Outcomes comparing GIC and non-GIC subgroups using linear mixed effects modeling.

Time	Change from Baseline *		Difference in Change, GIC vs. No GIC ^†^	*p*-Value
	No GIC	GIC		
**mJOA**				
Baseline	12.48	12.74		
1 year.	2.56	2.65	0.08 (-0.51 to 0.68)	0.787
2 year.	2.67	3.07	0.40 (-0.21 to 1.01)	0.201
**Nurick**				
Baseline	3.28	3.05		
1 year.	−1.40	−1.47	−0.07 (−0.38 to 0.24)	0.661
2 year.	−1.42	−1.65	−0.23 (−0.55 to 0.09)	0.164
**NDI**				
Baseline	38.23	46.31		
1 year.	−11.76	−13.36	−1.60 (−6.11 to 2.91)	0.488
2 year.	−12.36	−10.65	1.71 (−2.90 to 6.33)	0.468
**SF-36 PCS**				
Baseline	34.65	32.80		
1 year.	6.16	6.83	0.67 (−1.58 to 2.92)	0.561
2 year.	5.59	6.51	0.93 (−1.38 to 3.23)	0.432
**SF-36 MCS**				
Baseline	40.57	38.47		
1 year.	6.26	5.89	−0.37 (−3.35 to 2.61)	0.807
2 year.	5.94	4.52	−1.42 (−4.48 to 1.63)	0.361

* Values are reported as least-squares means of change in outcome scores from baseline at each follow-up time point. Baseline scores are reported as mean values for each treatment group. ^†^ Values are reported as difference in means (95% CI). Confidence intervals have not been adjusted for multiplicity.
